# Current News

**Published:** 1894-11

**Authors:** 


### Current News.

### THE HORACE WELLS ANNIVERSARY CELEBRATION.

Dear Doctor,—You are doubtless aware of the action of the American Dental Association at its recent meeting, held at Old Point Comfort, Virginia, with reference to holding a national celebration of the fiftieth anniversary of the discovery of the anæsthetic properties of nitrous oxide, by Dr. Horace Wells.

The committee, by vote of the American Dental Association, was instructed to secure two papers to be read at the celebration. One upon the "History of Anæsthesia," by Professor Thomas Fillebrown, of Boston, and one on the "Benefits of Anæsthesia to Mankind," by Professor James E. Garretson, of Philadelphia.

The committee was further instructed to arrange for a banquet to follow the meeting, at which distinguished speakers shall make appropriate addresses. A full report of the celebration, including the papers and addresses, to be printed and issued as a permanent souvenir of the occasion.

Arrangements have been completed to the extent of securing favorable responses from the essayists named, whose papers are now in course of preparation.

The banquet arrangements are also largely completed. To cover the expenses attending the celebration, the fee for admission to the banquet has been placed at six dollars. It is necessary that the committee shall have ample notice of the number who will be in attendance, in order that places may be provided for all who may desire to attend.

Subscriptions will be invited later for the souvenir volume, at a price sufficient to cover the cost of publication.

The celebration will be held in Philadelphia, on Tuesday, December 11, 1894.

You are cordially invited to participate in this event, which should enlist the enthusiastic support of every member of our profession.

To that end you are requested to send your check, and notify the chairman of the Anæsthesia Committee at the earliest date possible, in order that an official invitation may be sent to you.

It will be proposed at the meeting that subscriptions be invited for a permanent memorial, to take such shape as the meeting shall decide.

Signed,

LOUIS JACK, S. H. GUILFORD,

E. T. DARBY, WM. CARR,

C. N. PEIRCE, A. L. NORTHROP,

D. N. MCQUILLEN, H. B. NOBLE,

E. C. KIRK, JAS. MCMANUS.

J. D. THOMAS, *Chairman.*

912 WALNUT STREET, PHILADELPHIA.

---

### CHANGE OF NAME.

WE are requested to state that "The Oakland Chemical Co." has changed the name of their product from $H_2O_2$ to the true chemical name, Hydrogen Dioxide.

